# The outcome for surgical fixation of distal radial fractures in patients with idiopathic Parkinson’s disease: a cohort study

**DOI:** 10.1186/s13018-020-01642-5

**Published:** 2020-03-31

**Authors:** Te-Feng Arthur Chou, Chun Yao Chang, Chun-Ching Huang, Ming-Chau Chang, Wei-Ming Chen, Tung-Fu Huang

**Affiliations:** 1 Department of Orthopaedics and Traumatology, Taipei Veterans General Hospital, No. 201, Sec. 2, Shi-Pai Road, Taipei, 112 Taiwan, Republic of China; 2 Department of Orthopaedics, School of Medicine, National Yang-Ming University, No. 201, Sec. 2, Shi-pai Road, Beitou District, Taipei City, 11217 Taiwan, ROC; 3 Department of Exercise and Health, National Taipei University of Nursing and Health Science, No. 201, Sec. 2, Shi-Pai Road, Taipei, 112 Taiwan, Republic of China

**Keywords:** Parkinson’s disease, Distal radial fractures, Osteoporosis, Fracture nonunion

## Abstract

**Introduction:**

Idiopathic Parkinson’s disease (PD) is a progressive neurologic disorder causing postural instability and unsteady gait. These patients are at increased risk for fractures and have inferior outcomes after treatment. Several studies have evaluated the incidence and outcome of PD patients after hip fractures. However, there are limited studies assessing the outcome of upper extremity fractures in these patients. In this study, we evaluated the outcome of PD patients that received surgical intervention for distal radial fractures (DRF). We hypothesize that these patients have an inferior outcome after surgery in comparison with non-PD patients.

**Methods:**

Between May 2005 and May 2017, we retrospectively reviewed all of the patients with DRF and subsequently underwent open reduction and internal fixation (ORIF) at a level 1 trauma center. All of the surgeries were performed by fellowship-trained orthopedic surgeons. The inclusion criteria include patients with a definitive diagnosis of PD, non-pathological DRF, and a minimum follow-up of 1 year or up until the time of treatment failure was noted. Each PD patient was matched for age and gender to 3 non-PD patients. The primary objective was to determine the failure rate after surgical fixation for DRF. The secondary outcomes include time to treatment failure, reoperation rate, readmission rate, length of hospital stay, and postoperative complications.

**Results:**

A total of 88 patients were included in this study (23 PD, 65 non-PD patients). All underwent ORIF and received standard postoperative follow-ups. The overall treatment failure rate in PD was 39.1% vs. 4.6% in the non-PD group (*p* < 0.05). The time to treatment failure were 9.11 ± 3.86 weeks and 14.67 ± 5.8 weeks for PD and non-PD, respectively (*p* < 0.05). PD patients had a significantly higher rate of failure when k-wires and ESF were used (*p* < 0.05%), while loss of reduction was the most common mode of failure in PD (44.4%). The length of hospital stay for PD was 5.3 ± 4.69 days compared with 3.78 ± 0.96 days for non-PD (*p* = 0.01). There were 3 PD patients readmitted within 30 days after surgery, and 1 patient had pneumonia after the surgery.

**Conclusion:**

This study revealed that patients with PD have a high treatment failure rate despite surgical intervention for DRF. PD patients had a longer hospital stay and had a shorter time to treatment failure. In treating PD patients complicated with DRF, the surgeon must take into consideration the complex disease course of PD and the associated comorbidities such as osteoporosis, frail status, and frequent falls. Rehabilitation and disposition plans should be discussed in advance and longer hospital stays should be expected.

Level of evidenceLevel IV, retrospective cohort study

## Introduction

Idiopathic Parkinson’s disease (PD) is a chronic, progressive neurologic disorder characterized by rigidity, postural instability, and unsteady gait, leading to an increased risk for falls [1]. It is estimated that 70–87% of PD patients that are treated for longer than 20 years will have a fall injury [1]. In addition, it is estimated that these patients have almost two-folds of risk for fractures compared with the general population. The increased risk can be attributed to several factors. First, PD patients are at increased risks for falls and 90% of fractures in the elderly are directly related to falls [2]. Additional factors including advanced age, multiple comorbidities, and osteoporosis place PD patients at a significantly higher rate of fractures as well as postoperative complications [3]. On the other hand, several medication side effects also predispose PD patients to fractures. In particular, commonly used drugs such as monoamine oxidase-B (MAO-B) inhibitors, selective serotonin receptor inhibitors (SSRI), and high-dose antipsychotics all significantly increase the risk of osteoporotic fractures [4]. Therefore, it is essential for the physician to identify PD amongst this population in order to provide the appropriate care.

Of all types of fractures, the hip, forearm, and vertebrae are the most commonly affected sites [4]. Several studies have evaluated the outcome of PD patients after hip fractures [3]. Most of the literature suggests that PD is an independent risk factor for inferior outcomes after a hip fracture with increased risks for mortality, reoperation, surgical complications, and reduced mobility [5, 6]. Although hip fractures have been frequently discussed, limited studies have assessed the outcome of upper extremity fractures in PD patients. In particular, the distal radial fracture (DRF) is the second most common fracture observed in this population [4]. Since PD most often occurs in elderly patients, it can be intuitive to treat these patients with similar treatment methods as the elderly population. However, the appropriate treatment option for elderly patients that sustained a DRF remains controversial. The most recent American Academy of Orthopaedic Surgeons (AAOS) clinical practice guideline was unable to recommend for or against the treatment of distal radial fractures in elderly patients [7, 8]. If treated nonoperatively, the malunion rate can be as high as 89% [9]. For patients that received surgical intervention, poor wound healing, anesthesia related risks, and iatrogenic injuries are all concerns when treating the elderly [7]. With the significantly inferior outcome in the elderly, a comprehensive review to evaluate the outcome for DRF in PD is essential. In this study, we evaluated the outcome of PD patients that received surgical intervention for DRF and compared them with elderly patients without PD. We hypothesize PD patients who sustained DRF will have inferior outcomes compared with non-PD patients.

## Methods

This was a retrospective cohort study performed at a level 1 trauma center in Taipei, Taiwan. The study was approved by our hospital’s institutional review board. The primary outcome of the study was to determine the treatment failure rate of PD patients after surgical intervention for DRF. The secondary outcome was to determine the time to treatment failure, associated injuries, reoperation rate, length of hospital stay, readmission rate within 30 days, and postoperative complications (eg., pneumonia, UTI, delayed wound healing).

### Study population

The inclusion criteria were as follows: patients that underwent surgical fixation for a DRF with either fracture type A, B, or C by the OTA/AO classification system, patients that were 18 years old or older and were able to tolerate anesthesia, and had a minimum follow-up for 1 year or up to the time where treatment failure was noted. We excluded patients that declined surgery, under 18 years of age, and open or complex pathological fractures. The patients were allocated to either the PD group or to the non-PD group according to their diagnosis. A diagnosis of PD was made based on clinical history, review of medical records (ICD-9 CM code 332.0), and identifying PD-related drugs in the prescription records (levodopa-carbidopa, dopamine agonists, monoamine oxidase B inhibitors, catechol-O-methyltransferase inhibitors). A patient was assigned to the PD group if one of the above criteria for PD was met. If the patient did not meet our criteria, he/she was assigned to non-PD.

### Surgical intervention and patient assessment

All of the included patients underwent surgical reduction and fixation, either with plates, k-wires, external fixator (ESF), or a combination of the above methods. All of the surgeries were performed by fellowship-trained orthopedic surgeons. The choice of fixation method was determined based on the fracture pattern and the surgeon’s preference. All of the patients received a standard perioperative protocol. The medical records were comprehensively reviewed for pertinent histories such as American Society of Anesthesiologist physical status (ASA) grade, history of coronary artery disease (CAD), diabetes mellitus (DM), chronic kidney disease (CKD), osteoporosis, and smoking status. We evaluated each patient at 2 weeks, 1 month, 3 months, and 12 months after the surgery. If treatment failure was noted prior to the final follow-up, the follow-up duration was recorded as the time of treatment failure. Standard posteroanterior and lateral radiographs were obtained immediately after the surgery and at each visit. At each visit, we assessed the patient’s pain score with the visual analog scale (VAS). For radiographic assessment, we measured the radial inclination, articular step-off and palmar tilt for each visit. Treatment failure was considered if there was a loss of reduction, malunion or nonunion of the fracture. A loss of reduction is defined as a change in reduction in comparison to the immediate postop x-rays, including radial inclination > 5° of change, articular step-off > 2 mm, and palmar tilt > 5° of change. A malunion was defined as persistent pain upon activity (VAS > 5) 3 months after the surgery and/or limited radiographic evidence of fracture healing 6 months postoperatively. All measurements and assessments were completed by two senior orthopedic surgeons.

### Pilot study and patient population size

A pilot study consisting of 40 patients (20 patients in each group) was first completed to determine the ideal sample size required to achieve an alpha level of 0.05 with 80% power. The failure rate noted in the PD group and non-PD group were 35% and 3% respectively. We then matched our patients in a 1:3 ratio (PD:non-PD) for age and gender. These results indicate that a study consisting of at least 44 patients (12 patients:36 patients) was required to achieve statistical significance.

### Statistical analysis

All data were entered and analyzed with the SPSS software (version 25.0, SPSS Inc., Chicago, IL). We recorded data as mean, range, and standard deviation for continuous variables and the student’s *t* test was used to compare the differences at appropriate times. For categorical data such as percentages, we used the fisher’s exact test when the patient sample size was less than or equal to 5, and the chi-square test when the patient sample size was greater than 5 to assess for statistical significance. A *p* value < 0.05 and confidence interval > 95% were considered to be statistically significant.

## Results

After excluding the patients that did not meet the inclusion criteria, we enrolled a total of 88 patients in this study. There were 23 patients that had PD (mean disease duration 16.3 ± 2.1 months) with DRF and 65 non-PD patients with DRF. The mean follow-up duration was 8.83 ± 12.52 months.

### Baseline characteristics

The mean age was 75.7 ± 6.96 and 71.1 ± 8.81 years in the PD and non-PD group respectively (*p* = 0.11). The percentage of female patients were 73.9% in the PD group and 75.4% in the non-PD group (*p* = 0.89). The mean age, gender ratio, ASA grade, incidence of smoking, CAD, DM, and CKD were similar in both groups (Table 1). However, there was a significant difference in the fracture pattern between the groups. There was a significantly higher proportion of type C fractures (47.8% vs. 27.7%; PD:non-PD) for the PD patients, while there was a higher trend for type B fractures (4.3% vs. 27.7%; PD:non-PD) in the non-PD patients (*p* < 0.14). The baseline characteristics are shown in Table 1.

**Table 1:** Patient characteristics

	Overall	Patients w/ PD (*n* = 23)	Patients w/o PD (*n* = 65)	*p* value
Age (years)	72.35 ± 8.57	75.7 ± 6.96	71.1 ± 8.81	0.11
Female patients (%)	66 (75.0%)	17 (73.9%)	49 (75.4%)	0.89
Fracture type (AO/OTA)*				
Type A	40 (45.5%)	11 (47.8%)	29 (44.6%)	0.41
Type B	19 (21.6%)	1 (4.3%)	18 (27.7 %)	0.14
Type C	29 (33.0%)	11 (47.8%)	18 (27.7%)	0.67
ASA grade				0.78
Class I/II	9 (10.2%)	2 (8.7%)	7 (10.8%)	
Class III/IV	79 (89.8%)	21 (91.3%)	58 (89.2%)	
Smoking history	16 (18.2%)	5 (21.7%)	11 (16.9%)	0.61
Comorbidities				
CAD	13	3	10	0.54
DM	17	2	15	0.11
CKD	10	3	7	0.52
Osteoporosis	20	9	11	0.03
PD duration (months)	X	16.3 ± 2.1	X	X
Follow-up duration (months)	8.83 ± 12.52	6.57 ± 6.56	9.63 ± 14.0	0.18

*Fracture type was assessed using the AO/OTA classification 2007

*ASA* American Society of Anesthesiologist physical status classification system 2014, *PD* Parkinson’s disease, *CAD* coronary artery disease, *DM* diabetes mellitus, *CKD* chronic kidney disease

### Surgical method and outcome

**Fig. 1:**
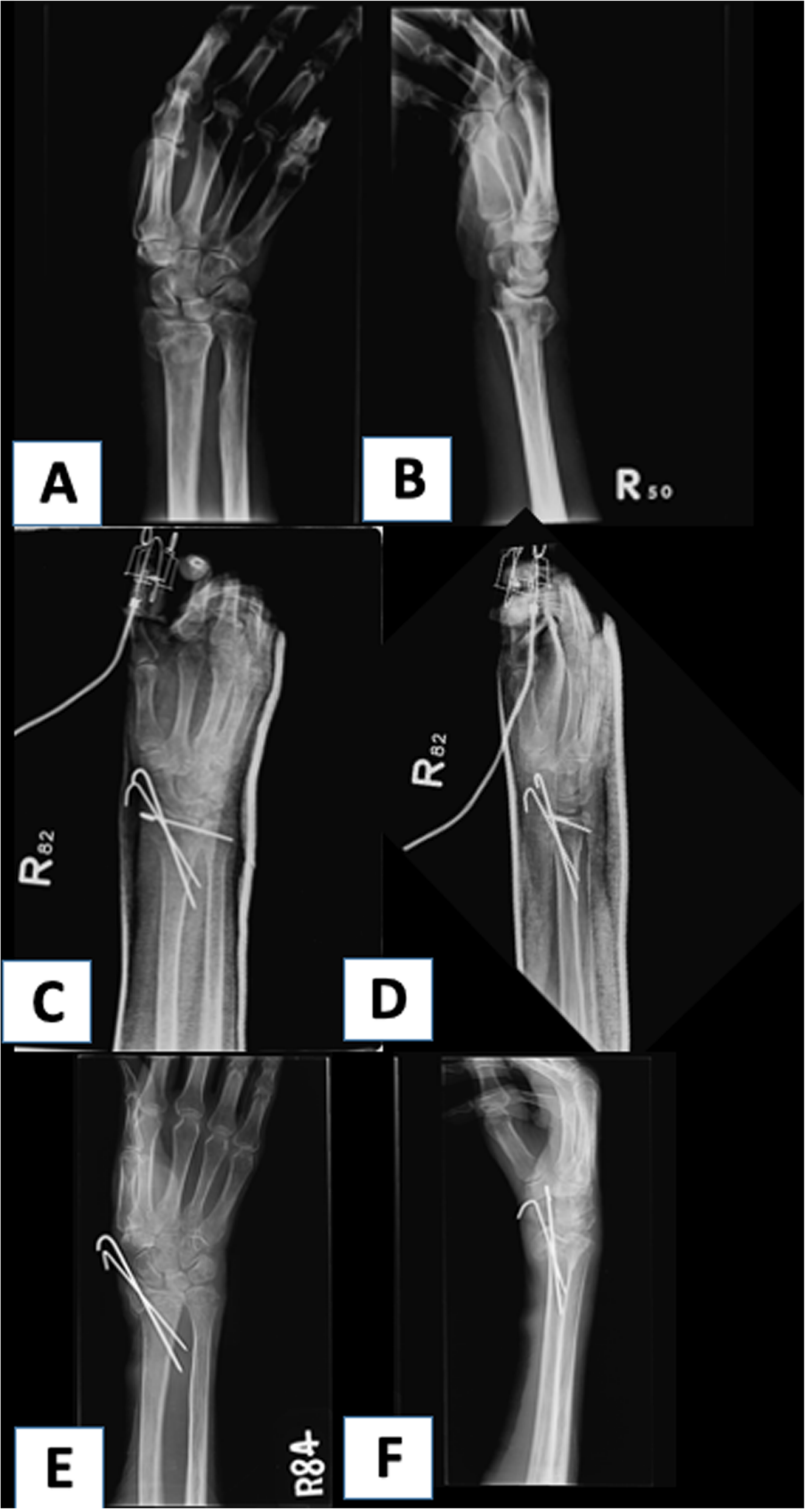
PD patient after k-wire fixation. This was a 67-year-old female that presented with right distal radial fracture and fixed with k-wires. **a** and **b** The preoperative radiographs. **c** and **d** Postoperative 2 weeks. **e** and **f** Postoperative 4 months showing loss of reduction

**Fig. 2:**
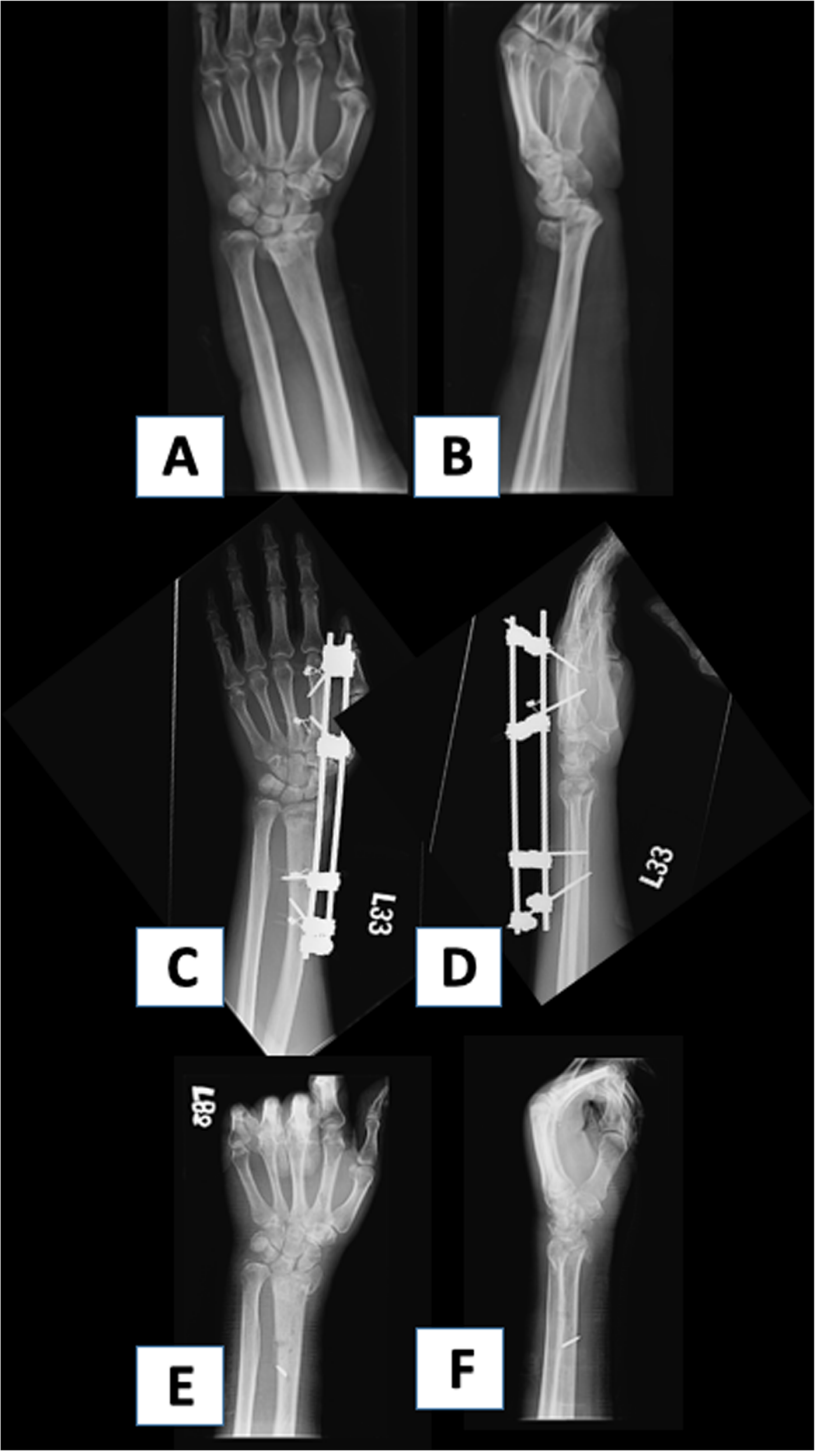
PD patient after ESF fixation. This was a 73-year-old female that presented with left distal radial fracture and fixed with ESF. **a** and **b** The preoperative radiographs. **c** and **d** Postoperative 3 days. **e** and **f** Postoperative 6 months status post removal of ESF with loss of reduction

The surgical method and outcome for both groups are shown in Table 2. The patients in PD had a higher percentage of k-wire fixation (26.1% vs. 6.2%), while a higher rate of plating was noted for the non-PD patients (56.5% vs. 78.5%), (*p* < 0.05). A total of 39.1% of the patients in the PD group had treatment failure at a mean follow-up of 6.57 ± 6.6 months, while the failure rate was 4.6% in non-PD at mean follow up of 9.63 ± 14.0 (*p* < 0.05). Two failure cases are presented in Figs. 1 and 2. In terms of choice of fixation, PD patients had a significantly higher rate of failure when k-wires and ESF were used (*p* < 0.05%), while plating of DRF appeared to have similar results between the two groups (Table 3). As for modes of failure, PD patients were more likely to have a loss of reduction and nonunion of fracture (17.4% and 13% vs. 1.5% and 1.5% for PD vs. non-PD respectively, *p* < 0.05). The length of hospital stay was 5.3 ± 4.69 days for PD and 3.78 ± 0.96 days for non-PD (*p* = 0.01). There was one PD patient who had a reoperation within 30 days due to screw penetration which required removal of internal fixation. In comparison, there were no reoperations for non-PD. In terms of 30-day readmissions, there were 3 patients from PD that were readmitted (one patient required removal of implant, another patient received treatment for postoperative pneumonia, and another patient had a second fall accident causing a hip fracture that required surgical fixation). One patient had a wound infection in non-PD which required debridement and was admitted for intravenous antibiotic treatment.

**Table 2:** Perioperative data

	Overall	Patients w/ PD	Patients w/o PD	*p* value
Number of patients	88	23	65	
Surgical intervention				
K-wires	10 (11.4%)	6 (26.1%)	4 (6.2%)	< 0.05
External Fixation	14 (15.9%)	4 (17.4%)	10 (15.4%)	0.53
Plates	63 (72.7%)	13 (56.5%)	51 (78.5%)	< 0.05
Outcome				< 0.05
Union	76 (86.4%)	14 (60.9%)	62 (95.4%)	
Treatment failure	12 (13.6%)	9 (39.1%)	3 (4.6%)	
Associated injuries	6 (6.8%)	3 (13%)	3 (4.6%)	0.17
Reoperation	1 (1.1%)	1 (4.3%)*	0	0.91
Length of stay (days)	4.42 ± 2.59	5.3 ± 4.69	3.78 ± 0.96	0.01
Readmission (< 30 days)	4 (4.5%)	3 (13.0%)	1 (1.5%)	0.05
Complications	2 (2.2%)	1 (4.3%)	1 (1.5%)	X
Pneumonia	1 (1.1%)	1 (4.3%)	0	
UTI	0	0	0	
Wound	1 (1.1%)	0	1 (1.5%)	
Mortality	0	0	0	
Thromboembolism	0	0	0	

*PD* Parkinson’s disease, *UTI* urinary tract infection

*Screw cutout leading to early removal of implant

**Table 3:** The modes of failure and time to failure

	Overall	Patients w/ PD	Patients w/o PD	*p* value
Treatment failure	12	9	3	< 0.001
K-wires	4	4	0	< 0.05
External fixation	4	3	1	< 0.05
Plates	4	2	2	0.28
Modes of failure				< 0.05
Loss of reduction	5 (41.6%)	4 (44.4%)	1 (33.3%)	< 0..05
Nonunion of fracture	4 (33.3%)	3 (33.3%)	1 (33.3%)	0.05
Persistent pain	3 (25%)	2 (22.2%)	1 (33.3%)	0.28
Time to failure (weeks)	10.5 ± 4.14	9.11 ± 3.86	14.67 ± 5.8	< 0.05

### Treatment failure and modes of failure

There were 39.1% of patients (*n* = 9) that had treatment failure in PD, and 4.6% (*n* = 3) in non-PD. The average time to treatment failure were 9.11 ± 3.86 weeks and 14.67 ± 5.8 weeks for the PD and non-PD group respectively (*p* < 0.05). The modes of failure are described in Table 3. For PD patients, loss of reduction was seen in 17.4% of patients, fracture nonunion in 13.0%, and persistent pain was observed after 6 months in 8.7% of patients.

## Discussion

To our knowledge, this was the first cohort study to evaluate the outcome of DRF in PD patients. The most significant finding of this study was the substantially higher failure rate noted in PD (39.1% vs. 4.6%; *p* < 0.05). In current literature, there are many studies that have indicated PD patients who have inferior outcomes and have increased risks for surgical complications after orthopedic procedures [3, 5, 10, 11].. Most studies have focused on hip fractures, and the outcome for DRF remains unknown.

### The outcome of fractures in patients with Parkinson’s disease and elderly patients

Although the majority of patients that receive surgical fixation for DRF have good to excellent outcomes, PD patients appear to have inferior results. In the general population, the nonunion rate is reported to be as low as < 1% and the overall complication rate in current literature for DRF ranges from 14 to 30% in the general population [4, 12–14]. In our study, we considered patients to have a treatment failure if there was loss of reduction, malunion or nonunion of fracture, or persistent pain at the injured site 6 months after surgery. We noted 4.6% of the non-PD patients had treatment failure, while 39.1% of the patients in the PD group were considered to have treatment failure. The cause for this substantially higher rate of treatment failure is currently unknown, but it is most likely multifactorial. For instance, PD patients often exhibit severe rigidity which may cause early failure of fixation [15]. Hence, there was a significantly higher rate of loss of reduction in the PD group. In addition, the frail status and frequent falls exhibited in this patient population may further complicate the postoperative recovery course [6]. Lastly, the frequent resting tremors observed in PD may also have altered the healing process. In order to facilitate optimal bone healing to occur, stabilizing the fracture either through relative stability or absolute stability is essential [16]. Most of the current studies for fractures in PD patients have focused on hip fractures. Roche et al. evaluated 2448 patients that sustained a hip fracture, 97 of whom had PD. Their results suggested PD was not a risk for 1-year mortality. On the other hand, several reports noted an inferior outcome for PD patients after sustaining a hip fracture with mortality rates as high as 47% [6, 17]. Karadsheh et al. reported PD as an independent predictor of mortality after operative treatment for femoral neck fractures. Interestingly, PD patients were more likely to sustain a dislocation after hemiarthroplasty and fixation failure for minimally displaced fractures [6]. The reoperation rates for PD were 4% and 22% for displaced femoral neck fractures and nondisplaced fractures respectively. Although the two groups received different surgical interventions, the significantly higher reoperation rates in the nondisplaced group prompted the authors to favor the use of hemiarthroplasties for non-displaced femoral neck fractures in PD patients.

### The choice of fixation method for DRF in PD patients

Although k-wire fixation and ESF are often considered to be relatively stable fixation techniques and provide fairly good outcomes in the general population, it should be used with caution in PD. In this study, there was a higher percentage of patients undergoing k-wire or ESF fixation in PD (*n* = 10, 43.5% vs. *n* = 14, 21.6%; for PD and non-PD respectively). For patients that underwent k-wire fixation, 4 (66.7%) PD patients had treatment failure while none were observed in the non-PD group (*n* < 0.05). Meanwhile, 3 (75%) patients that received ESF had treatment failure while only 1 patient in non-PD (10%) had a treatment failure (*p* < 0.05) (Table 3). The higher rate of failure is currently unknown. However, PD patients often have frequent tremors and may cause micro-instability over the fracture site and may impede proper fracture healing. In addition, the constant rigidity exhibited in these patients may also cause excessive muscle contracture, leading to displacement of the fracture fragments. In agreement with our results, we noted the most common modes of failure were loss of reduction and nonunion of fracture in PD patients. Therefore, our authors recommend a rigid fixation method such as locked plates for PD patients in order to assure proper fracture healing.

### Length of hospital stay and potential problems in caring PD patients

PD patients often have multiple comorbidities which may complicate their postoperative care. Pouwels et al. identified cancer, ischemic heart disease, and cerebrovascular disease as the three most common associated comorbidities in PD patients that have sustained a fracture [4]. In this study, we noted osteoporosis to be the most commonly associated disease in a relatively small sample size, which can partly explain the higher incidence of osteoporotic fractures. In a comprehensive review of 9225 patients with hip fracture (452 patients with PD), Coomber et el. noted a significantly longer hospital stay for PD patients [3]. In-hospital complications were relatively common, of which postoperative delirium, pressure sores, pneumonia all occurred more often in patients with PD. In this study, PD patients were admitted in an acute orthopedic ward for 5.3 ± 4.69 days while non-PD patients had a shorter stay of 3.78 ± 0.96 days (*p* = 0.01). There was one PD patient complicated with aspiration pneumonia which required extended stay for treatment. Most of the postoperative complications recorded in literature can be partly attributed to the disease nature of PD as well as medication side effects (eg., sedatives). Therefore, we routinely consult neurology and geriatric specialists to manage the postoperative course.

For patients in the non-PD group, only one patient had wound dehiscence which was treated in under local anesthesia with debridement and primary closure of the wound. This patient was subsequently admitted for intravenous antibiotics. As for 30-day readmissions, there was a significantly higher rate of readmissions for PD (13% vs. 1.5%, *p* = 0.05). One patient required removal of implant, another patient received treatment for postoperative pneumonia, and another patient had a hip fracture that required surgical fixation. In addition to proper management of the DRF, detailed workup for osteoporosis and fall prevention measures are essential for PD patients to reduce postoperative complications. Since the bone mineral density in patients with PD is lower compared with healthy controls, appropriate medications such as bisphosphonates and denosumab should be initiated once osteoporosis is confirmed [18].

There are several limitations in this study. This was a retrospective medical record review which may have certain bias due to the nature of the study design. In addition, there was a relatively small sample size for PD patients. In order to determine the adequate sample size, we conducted a pilot study to assure statistical significance was achieved. Furthermore, we matched our patients for age and gender in a 1 to 3 ratio with non-PD patients to overcome the relatively small sample size. Another limitation is that we did not assess the clinical function and PD disease status of our patients which could provide a better overall evaluation. Finally, future studies should also include a group of PD patients that received nonoperative management after DRF to better assess the necessity of surgical fixation in this unique group.

## Conclusion

In PD patients with a distal radial fracture, there was higher treatment failure rate in comparison to the non-PD group. PD patients tend to have longer hospital stays and a shorter time to treatment failure. Although internal fixation of DRF can be done with different instruments, we recommend plating of the radius over k-wire fixation for better outcomes. The treating physician must also take into account the complex disease course of PD and manage comorbidities such as osteoporosis, frail status, and unintentional tremors accordingly. Rehabilitation and disposition plans should be discussed in advance and longer hospital stays should be expected.

## Data Availability

not applicable

## Abbreviations

PD: Parkinson’s disease
DRF: Distal radial fractures
AAOS: American Academy of Orthopaedic Surgeons
ORIF: Open reduction and internal fixation
ESF: External fixator
ASA: American Society of Anesthesiologist
VAS: Visual analog scale
K-wires: Kirschner wires
